# Hypoxia-inducible factor in breast cancer: role and target for breast cancer treatment

**DOI:** 10.3389/fimmu.2024.1370800

**Published:** 2024-05-10

**Authors:** Shijiao Zhi, Chen Chen, Hanlin Huang, Zhengfu Zhang, Fancai Zeng, Shujun Zhang

**Affiliations:** Department of Biochemistry and Molecular Biology, School of Basic Medical Sciences, Southwest Medical University, Luzhou, Sichuan, China

**Keywords:** breast cancer, hypoxia-inducible factor, angiogenesis, invasion and metastasis, apoptosis and autophagy, drug resistance

## Abstract

Globally, breast cancer stands as the most prevalent form of cancer among women. The tumor microenvironment of breast cancer often exhibits hypoxia. Hypoxia-inducible factor 1-alpha, a transcription factor, is found to be overexpressed and activated in breast cancer, playing a pivotal role in the anoxic microenvironment by mediating a series of reactions. Hypoxia-inducible factor 1-alpha is involved in regulating downstream pathways and target genes, which are crucial in hypoxic conditions, including glycolysis, angiogenesis, and metastasis. These processes significantly contribute to breast cancer progression by managing cancer-related activities linked to tumor invasion, metastasis, immune evasion, and drug resistance, resulting in poor prognosis for patients. Consequently, there is a significant interest in Hypoxia-inducible factor 1-alpha as a potential target for cancer therapy. Presently, research on drugs targeting Hypoxia-inducible factor 1-alpha is predominantly in the preclinical phase, highlighting the need for an in-depth understanding of HIF-1α and its regulatory pathway. It is anticipated that the future will see the introduction of effective HIF-1α inhibitors into clinical trials, offering new hope for breast cancer patients. Therefore, this review focuses on the structure and function of HIF-1α, its role in advancing breast cancer, and strategies to combat HIF-1α-dependent drug resistance, underlining its therapeutic potential.

## Graphical Abstract

Schematic overview of HIF-1a regulation in breast cancer. Under normoxic conditions, HIF-1α is unstable with a brief half-life. In hypoxic conditions, the depletion of molecular oxygen during mitochondrial oxidative phosphorylation curtails the catalytic functions of PHD and FIH, limiting HIF-1α hydroxylation and degradation, thereby activating the HIF pathway. Subsequently, HIF-1α is involved in angiogenesis, glucose metabolism, cancer cell invasion and metastasis, and immune escape by regulating the expression of target genes.

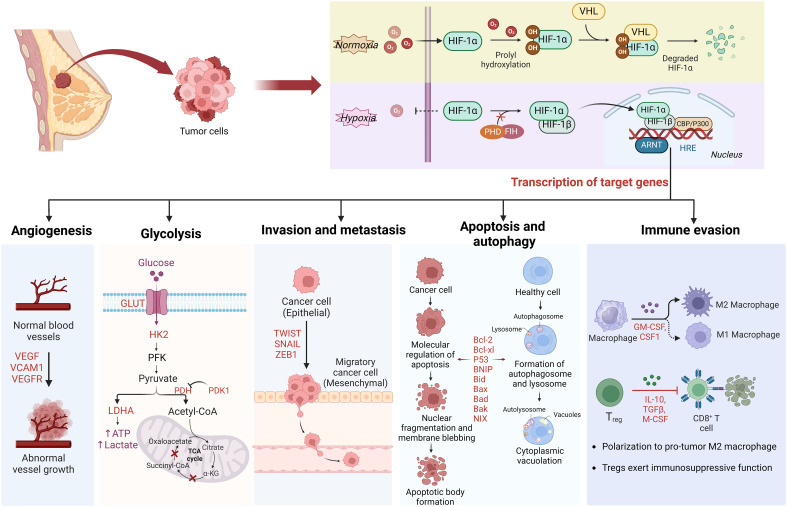

## 1 Introduction

Breast cancer (BC) is the most common type of malignancy among women and the second leading cause of cancer-related deaths among women after lung cancer (1–3). The treatment of breast cancer mainly includes surgery, endocrine therapy, chemotherapy, radiotherapy, and targeted therapy, depending on the classification of the tumor, with drug therapy playing an important role. In earlier years, the mortality rate of breast cancer patients has declined due to the reduced risk of the disease, improved treatment methods, and the widespread use of early screening (4). However, the emergence of drug resistance during treatment in recent years has posed a severe challenge to the survival of breast cancer patients (5). Hypoxia, caused by an imbalance between oxygen consumption and supply due to rapid tumor growth, is a common feature of the tumor microenvironment in most solid tumors (4, 6). It promotes tumor growth, metastasis, and treatment resistance by regulating the expression of hypoxia-related genes, ultimately leading to more aggressive and fatal cancers. Hypoxia is associated with enhanced invasive behavior and poorer prognosis and has been identified as a poor indicator of patient outcome (7). The hypoxia-inducible factor (HIF) family, which plays a pivotal role in the cellular response to hypoxic stress, consists of transcription factors that are crucial for managing hypoxic stress at the cellular level (8, 9). Research has consistently demonstrated that Hypoxia-inducible factor 1-alpha (HIF-1α) is overexpressed in numerous types of cancer, significantly influencing cancer progression (10–16). HIF-1α is responsible for activating genes associated with angiogenesis, cell growth and survival, invasion and metastasis, glucose metabolism, immune system evasion, and resistance to several cancer therapies. Specifically, HIF-1α is involved in regulating tumor cell metabolism, apoptosis, and autophagy, thereby impacting their survival (8, 17–22). It represents a promising target for anticancer therapy. Therefore, this article provides a detailed description of the structure and function of HIF-1α as well as its mechanism of action in the development of breast cancer. This review also summarizes the reasons for the emergence of HIF-1α-dependent drug resistance and strategies to overcome it, providing systematic information for the development of targeted drugs against HIF-1α.

## 2 Structure and function of HIF-1α

Hypoxia significantly influences numerous pathophysiological conditions in the human body (1). It is a defining characteristic of the solid tumor microenvironment (TME), resulting from rapid tumor growth and inadequate blood supply. An estimated 50%–60% of tumors exhibit anoxic regions (23). Hypoxia is linked to cancer spread and resistance to conventional therapies like chemotherapy and radiotherapy, indicating a grim prognosis for various cancers, including BC, hepatocellular carcinoma (HCC), and cancers of the pancreas, stomach, and colorectum (24). Therefore, targeting hypoxia is seen as a viable strategy in cancer treatment. Cells have evolved sophisticated mechanisms to adapt to anoxic conditions (25).

Within the hypoxic TME, HIF plays a crucial role in tumor adaptation (26). To date, mammals have been found to express three HIF types. HIF is a heterodimer consisting of an oxygen-sensitive α subunit (HIF-1α, HIF-2α, and HIF-3α) and an oxygen-insensitive β subunit (HIF-1β), crucial for regulating gene expression under hypoxic conditions (27–29). While oxygen levels regulate all three HIF α subunits and they all bind to HIF-1β, research has primarily focused on HIF-1α and HIF-2α (29–34). HIF-1α and HIF-2α share structural similarities, yet their roles differ across tumor and cell types (29, 35, 36). HIF-3α has been less studied due to its multiple variants and complex functionality ( (37, 38). Recent research indicates that specific HIF-1α subtypes are present in several solid tumors, potentially contributing to tumor progression (1, 39).

**Figure 1:**
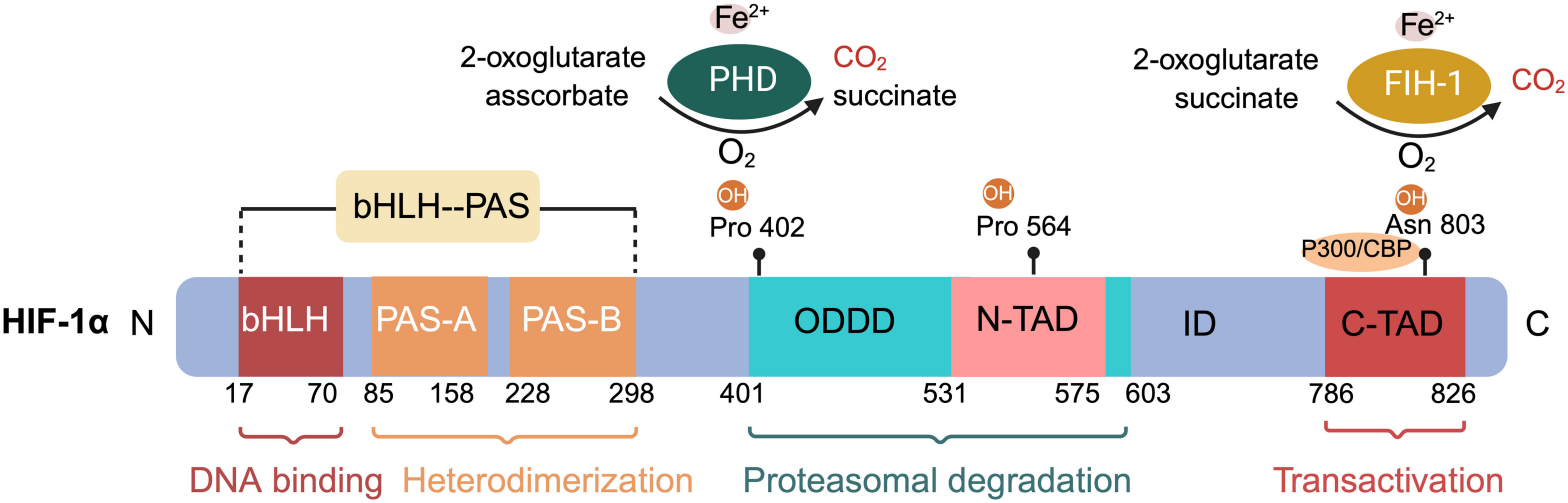
Schematic of hypoxia-inducible factor HIF-1α protein structure and hydroxylation sites at proline and asparagine residues. The basic submotif and the helix-loop-helix domain (bHLH) are located near the N terminus, followed by the Per-ARNT-Sim (PAS) domain. The PAS domain comprises repetitive amino acid sequences PAS-A and PAS-B. The oxygen-dependent degradation domain (ODDD) overlaps with the N-terminal transactivation domain (N-TAD), followed by the C-terminal transactivation domain (C-TAD). Hydroxylation of proline residues within the ODDD and of asparagine residues within the C-TAD of HIF-1α are highlighted. The non-equilibrium hydroxylation by the prolylhydroxylases (PHD) and the asparagine hydroxylase factor inhibiting HIF-1 including substrates and products is depicted exemplarily for two of the three hydroxylation sites of HIF-1α.

HIF-1 consists of the HIF-1α subunit, composed of 826 amino acids, and the HIF-1β subunit, which has 782 amino acids (**Figure 1**) (40, 41). HIF-1α is the most extensively expressed subunit of HIF-1 in mammalian tissues (8). This transcription factor, encoded by the HIF-1α gene on chromosome 14q2124, responds to hypoxic signals (42, 43). It is part of the basic helix–loop–helix (bHLH)/Period Clock Protein (Per)–Aryl Hydrocarbon Receptor Nuclear Translocator (ARNT)–Single-minded Protein (Sim) (bHLH/PAS) family of transcription factors (44). The bHLH and PAS domains, named after the proteins Per, ARNT, and Sim (40) first identified in Drosophila, are essential for DNA binding and the formation of heterodimers between HIF-1α and HIF-1β, respectively (45). HIF-1α includes two transactivation domains (TAD): N-TAD and C-TAD, enriched with acidic and hydrophobic amino acids, linked by an inhibitory domain (ID) (42). C-TAD plays a role in HIF-1α transcription regulation through interaction with the transcriptional coactivator CREB-binding protein (CBP)/p300 in hypoxic conditions, while N-TAD serves as its stable regulator (46, 47). The ID, situated between the two TAD sequences (amino acids 576–785), prevents transcriptional activation by TADs (48). Furthermore, HIF-1α features an oxygen-dependent degradation domain (ODDD) upstream of the N-TAD region, including two hydroxylation sites, Pro-402 and Pro-564, each bearing a conserved LXXLAP motif (49). This ODDD, located centrally in HIF-1α, chiefly mediates the protein’s oxygen-regulated stability and degradation through the ubiquitin-proteasome pathway (50, 51). HIF-1α also possesses two nuclear localization signals (NLS), NLSN (N-terminal, 17–33 amino acids) and NLSC (C-terminal, 718–721 amino acids) (45). HIF-1β, also known as ARNT, is expressed constitutively in all cell types and its expression is not influenced by oxygen levels (52). It includes three domains: bHLH, PAS, and C-TAD, but lacks the ODDD and N-TAD domains (45, 53).

**Figure 2:**
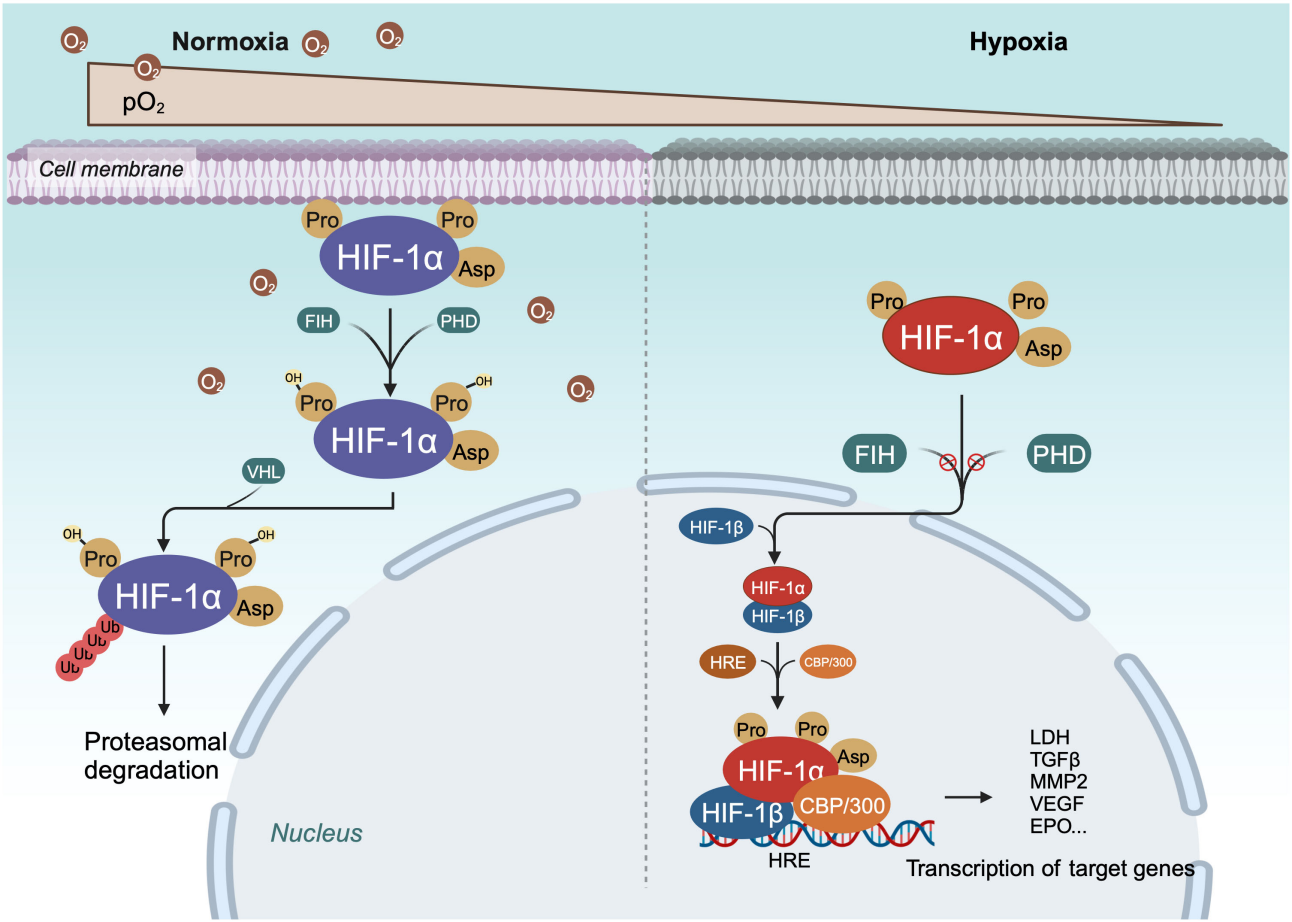
Schema of regulation of HIF-α degradation and transcriptional activity. Under normoxic conditions, HIF-1α is continuously degraded through the key oxygen sensor PHD, which enables HIF-1α to bind to VHL. Under hypoxic conditions, the hydroxylation of HIF - 1α is inhibited, leading to stabilization of HIF-1α. Next, HIF-1α dimerizes with HIF-1β to form a transcriptional activation complex, which binds to HRE and stimulates the transactivation of target genes.

Within cells, the regulation of HIF-1α is stringently dependent on oxygen availability, in contrast to HIF-1β, which is constantly expressed irrespective of oxygen tension (40, 51). Under normoxic conditions, HIF-1α is unstable with a brief half-life (**Figures 1**, **2**) (40). Pro-402 and Pro-564 in the ODDD and Asn-803 in the C-TAD are hydroxylated by prolyl hydroxylases (PHD) and factor-inhibiting HIF (FIH) using Fe^2+^ and 2-oxoglutaric acid as cofactors (40, 51). Hydroxylation at Asn-803 inhibits CBP/p300 binding to HIF-1α, while hydroxylation at proline residues allows the von Hippel-Lindau tumor suppressor protein (pVHL), with E3 ubiquitin ligase activity, to recognize hydroxylated HIF-1α (42, 54). Consequently, HIF-1α is ubiquitinated and swiftly degraded through the pVHL-mediated ubiquitin-proteasome pathway. The second regulation mechanism involves the hydroxylation of asparaginyl residues by FIH (**Figures 1**, **2**) (55), preventing the association of HIF coactivators (CBP/p300) (55, 56). This asparaginyl hydroxylation occurs in the C-TAD domain at N803 in HIF-1α and N851 in HIF-2α (57). Thus, under normoxic conditions, PHD and FIH facilitate HIF degradation through a dual mechanism, suppressing HIF transcriptional activity (58). In hypoxic conditions, the depletion of molecular oxygen during mitochondrial oxidative phosphorylation curtails the catalytic functions of PHD and FIH (**Figure 2**) (57, 59), limiting HIF-1α hydroxylation and degradation, thereby activating the HIF pathway (60, 61). The stable HIF-1α then moves to the nucleus and forms a transcriptionally active heterodimer with HIF-1β (29). The interaction occurs between the heterodimeric complex of HIF-1α/HIF-1β and CBP/p300, the steroid receptor coactivator-1 family of coactivators, the nuclear redox regulator Ref-1, and the molecular chaperone heat shock protein 90. This interaction facilitates the binding of hypoxia response elements (HRE), leading to increased transcriptional activity of target genes across various signaling pathways and the regulation of cellular adaptive responses to hypoxia (**Table 1**) (29, 78, 79). However, the degradation of accumulated HIF-1α happens rapidly upon reoxygenation of hypoxic cells, with the rate of degradation being dependent on the duration of hypoxic stress (80).

**Table 1:** Target genes of the HIF-1α and their function.

Target Gene	Expression under hypoxia	Function	Reference
GLUT1	Up	Glycolysis	(62)
LDHA	Up	Glycolysis	(62)
PKM2	Up	Glycolysis	(62)
LOX	Up	Invasion	(63)
SOD2	Up	Enrichment of braest cancer stem cell	(64)
A2BR	Up	Invasion, and metastasis	(65)
MDR1	Up	Enrichment of braest cancer stem cell	(62)
VEGF	Up	Angiogenesis	(66)
Bcl-2	Up	Apoptosis	(67)
GLUT	Up	Glycolysis	(68)
NANOG	Up	Invasion	(69)
PDK 1	Up	Glycolysis	(68)
IL-6	Up	Invasion	(70)
IL-8	Up	Invasion	(71)
MMPs	Up	Invasion	(71)
NF-κB,	Up	Invasion	(72)
HK2	Up	Glycolysis	(73)
C1QBP	Up	Angiogenesis	(74)
P4HA2	Up	Invasion and metastasis.	(75)
SLUG	Up	Plays a key role in the control of epithelial to mesenchymal transition	(76)
TWIST	Up	Plays a key role in the control of epithelial to mesenchymal transition	(77)

## 3 The role of HIF-1α in breast cancer

### 3.1 Angiogenesis

BC cells necessitate a continual blood supply for oxygen and essential nutrients (81). Initially, during tumor growth, nutrients and oxygen are obtained via diffusion (82). However, as the tumor mass reaches a certain size, diffusion becomes insufficient to sustain growth, prompting the formation of new vasculature to support tumor growth and metastasis (81, 83). Thus, angiogenesis is critical for tumor advancement, proliferation, and metastasis. Tumor blood vessels exhibit high tortuosity, increased vascular permeability, and sluggish blood flow (62)compared to normal vessels, leading to increased local hypoxia, which in turn stabilizes HIF-1α, fostering tumor invasion and metastasis (84–87)

**Figure 3:**
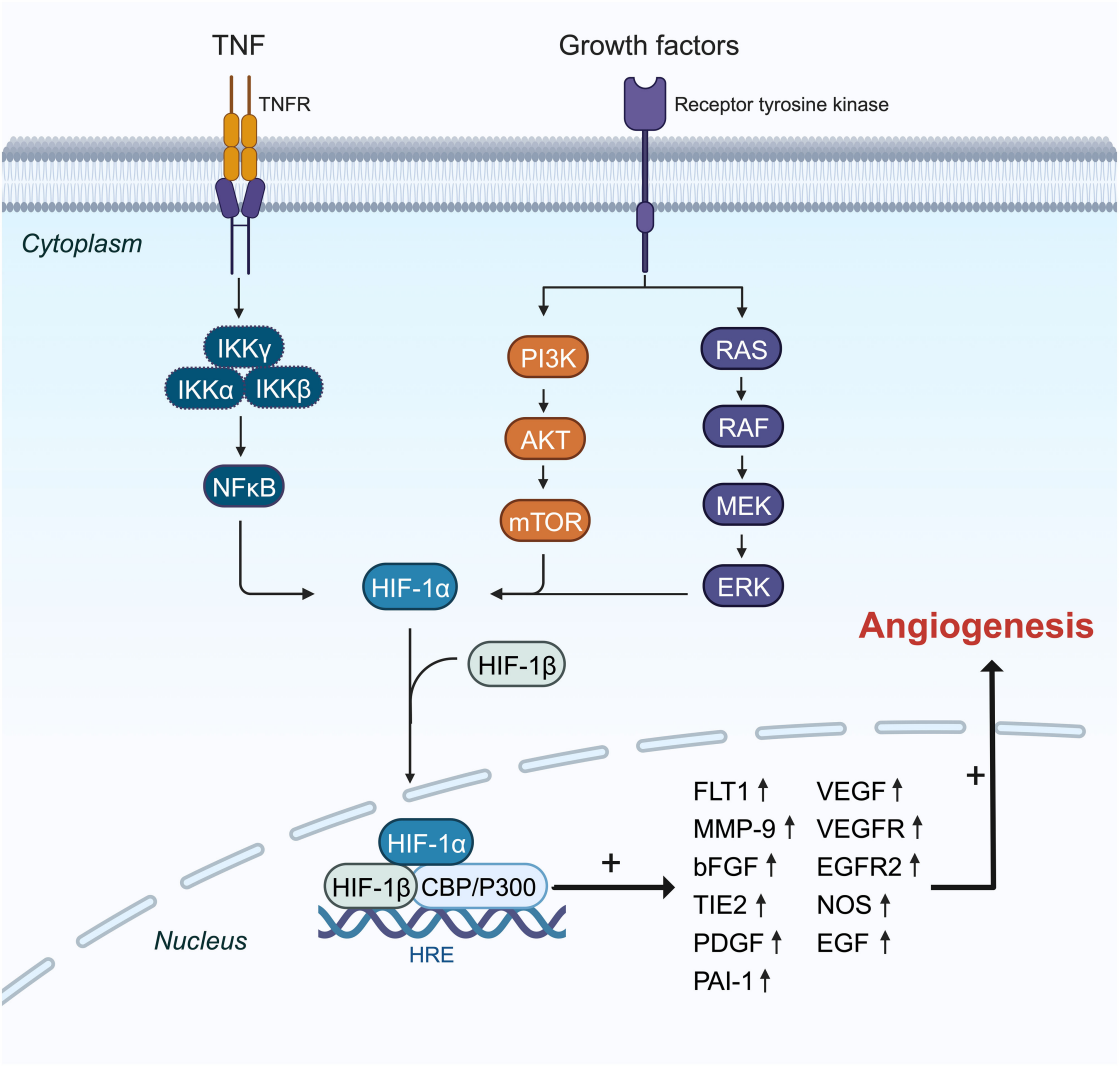
The mechanisms and pathways of HIF-1α overexpression effects on the induction of angiogenesis genes. HIF-1α translation is amplified by PI3K, RAS, and NF-κβ pathways in the cytoplasm, and then it can pass into the nucleus with coactivator (P300) as a transcription factor and enhances the expression of some essential angiogenesis genes like FLT1, MMP9, VEGF, VEGFR, and PAI-1 in the angiogenesis process.

HIF plays a pivotal role in regulating angiogenesis (**Figure 3**) (88, 89). BC cells exhibit elevated levels of HIF, stimulating gene expression that facilitates proliferation, metastasis, angiogenesis, and invasion (90, 91). HIF-1α is notably expressed in precursor lesions and early stages of BC (92). Suppression of the HIF-1α gene or inhibition of its transcription can hinder tumor cells from secreting vascular endothelial growth factor (VEGF) and impede tumor neovascularization (8, 93, 94).

HIF-1α shows increased expression particularly in triple-negative BC (TNBC). The nuclear factor kappa B (NF-κB) signaling pathway is activated in TNBC, promoting tumor cell proliferation, angiogenesis, and evasion of apoptosis (**Figure 3**) (95). Tumor necrosis factor-alpha (TNF-α) or interleukin (IL)-1 activates inhibitory kappa B kinase (IKKB), leading to NF-Kb activation (96). Additionally, under chronic hypoxia in TNBC cell lines, reactive oxygen species (ROS) further activate NF-κB by degrading inhibitor of κB-α (IκBα) (97). The NF-κB pathway boosts HIF-1α mRNA expression by enhancing its transcription (98, 99). TNF-α may elevate HIF-1α expression by activating NF-κB signaling in TNBC cells and could be influenced by IL-17 (100).

In BC, HIF-1α primarily induces angiogenesis by regulating the expression of VEGF, hepatocyte growth factor (HGF), vascular cell adhesion molecule 1 (VCAM1), and VEGF receptor (VEGFR) (**Figure 3**) (62, 101). VEGF, a critical downstream target of the HIF pathway, drives angiogenesis by influencing endothelial cell migration, proliferation, permeability, and survival (102–105). Under hypoxic conditions, HIF-1α binds to the VEGF promoter, significantly increasing VEGF mRNA levels in TNBC compared to other BC subtypes (106). HIF-1α also upregulates breast tumor kinase mRNA and protein expression, which stimulates angiogenesis via hepatocyte growth factor (107). Furthermore, HIF-1α induces overexpression of C1q binding protein (C1QBP), indirectly stimulating the NF-κB signaling cascade to upregulate VCAM1 expression and promote TNBC angiogenesis (74). Short-term hyperoxia induces ROS formation, leading to increased brain-derived neurotrophic factor expression and VEGFR receptor upregulation through HIF-1α, promoting angiogenesis (108).

Although HIF-1α predominates in BC, the HIF-2α isoform is equally crucial as a key regulator of pathophysiological angiogenesis (109). *RAB11B-AS1*, a long noncoding RNA (lncRNA), enhances VEGFA and angiopoietin-like 4 (ANGPTL4) expression in hypoxic BC cells in a HIF-2α-dependent manner, promoting tumor angiogenesis and metastasis (110). In conclusion, angiogenesis is a multifaceted process, and HIF-1α significantly contributes to angiogenesis in BC (62, 111).

### 3.2 Glucose metabolism

**Figure 4:**
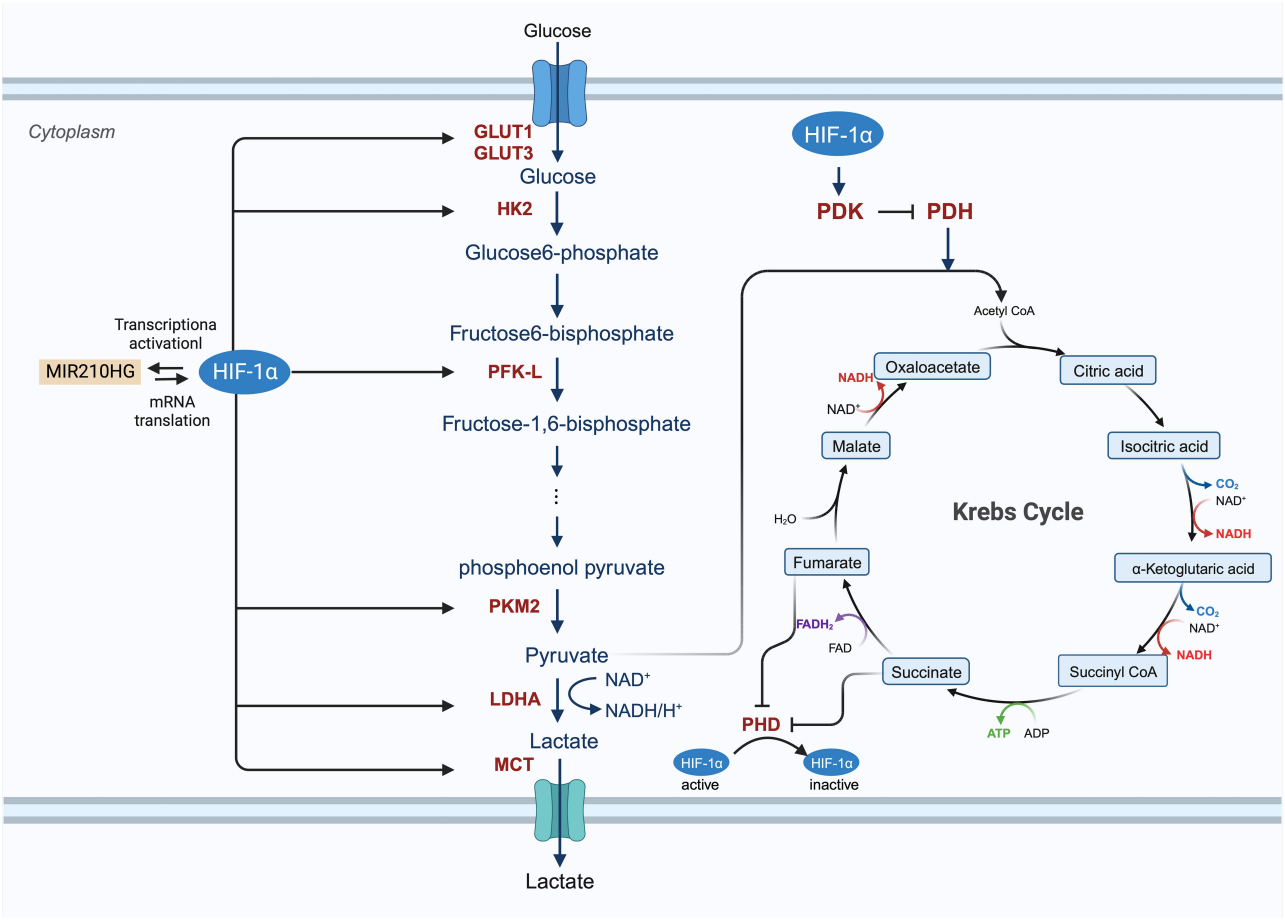
The involved procedure of HIF-1α in cancer glucose metabolism. HIF-1 enhances the expression of glucose transporters GLUT1 and GLUT3 and activates glycolytic enzymes, including hexokinase 2 (HK2), phosphofructokinase (PFK-L), pyruvate kinase isozymes M2 (PKM2) to generate an increasing amount of pyruvate. After this process, pyruvate is largely converted to lactate by lactate dehydrogenase A (LDHA) and removed from the cancer cell by monocarboxylate transporter (MCT). HIF-1 also inhibits the TCA cycle and oxidative phosphorylation process by activating the expression of HIF-1-dependent pyruvate dehydrogenase kinase (PDK), resulting in the decrease of mitochondrial activities and the oxygen consumption in hypoxia.

Enhanced glucose metabolism plays a vital role in the growth and division of cancer cells, which require significant amounts of biomass and alternate energy sources to offset the diminished oxidative phosphorylation. The activity of HIF is believed to be intimately associated with glucose metabolism. Genes activated by HIF transcription include crucial components of glucose metabolism, such as glucose transporters (GLUT1 and GLUT3), enzymes involved in the glycolytic pathway (hexokinase, phosphofructokinase, aldolase, glyceraldehyde 3-phosphate dehydrogenase, phosphoglycerate kinase, enolase, pyruvate kinase, and lactate dehydrogenase [LDH]), and pyruvate dehydrogenase (PDH) kinase (PDK) (**Figure 4**) (28, 55, 112, 113). Du et al. investigated the potential roles and mechanisms of the hypoxic *MIR210HG* axis, finding that *MIR210HG* increases HIF-1α protein levels by directly interacting with the 5’-UTR of HIF1α mRNA. This elevation in HIF-1α protein enhances the expression of enzymes related to glycolysis (pyruvate kinase M2, LDHA) and GLUT1 (114). In hypoxic conditions, HIF-1α facilitates the shift of tumor cells from oxidative to glycolytic metabolism by triggering genes that encode glucose transporters and glycolytic enzymes (115). In particular, the overexpression of HIF-1α in glucose augments glucose absorption in tumor cells through the elevation of glucose transporter levels. The modification of proteins by O-linked β-N-acetylglucosamine (O-GlcNAc) (O-GlcNAcylation) influences glycolysis in BC cells via the HIF-1α/GLUT1 signaling pathway (81, 116). Glucose is processed through the glycolytic pathway in tumor cells, generating substantial amounts of pyruvate (117). Hexokinase 2 (HK2) serves as both the initial and rate-limiting enzyme in the glycolysis pathway (81). CircRNF20 is found to accelerate tumor progression by targeting miR-487a/HIF-1α/HK2 in BC (73). HIF-1α enhances PDK activity and blocks the transformation of pyruvate to acetyl-CoA by inhibiting PDH; thus, reducing the entry of pyruvate into the tricarboxylic acid cycle (55, 118). LDHA converts pyruvate into lactic acid, which is then transported out of the cell by the monocarboxylate transporter (MCT) (62). As cancer cell metabolism shifts to aerobic glycolysis, lactate supplants pyruvate and is expelled into the tumor microenvironment (TME), thereby fostering an immunosuppressive milieu that supports tumor cell proliferation, metastasis, and invasion (119, 120).

Glycolysis can lead to an increase in HIF-1α levels, which in turn raises VEGF expression (121). Aerobic glycolysis stimulates angiogenesis by producing lactate, which acidifies the extracellular environment and enhances VEGF expression (122). Additionally, the end products of glycolysis, lactate and pyruvate, influence VEGF expression through the augmentation of HIF-1α levels (123–125). Hence, it is postulated that the transition to glycolysis precedes angiogenesis. In summary, HIF-1α plays a crucial role in regulating glucose metabolism in BC.

### 3.3 Invasion and metastasis

**Figure 5:**
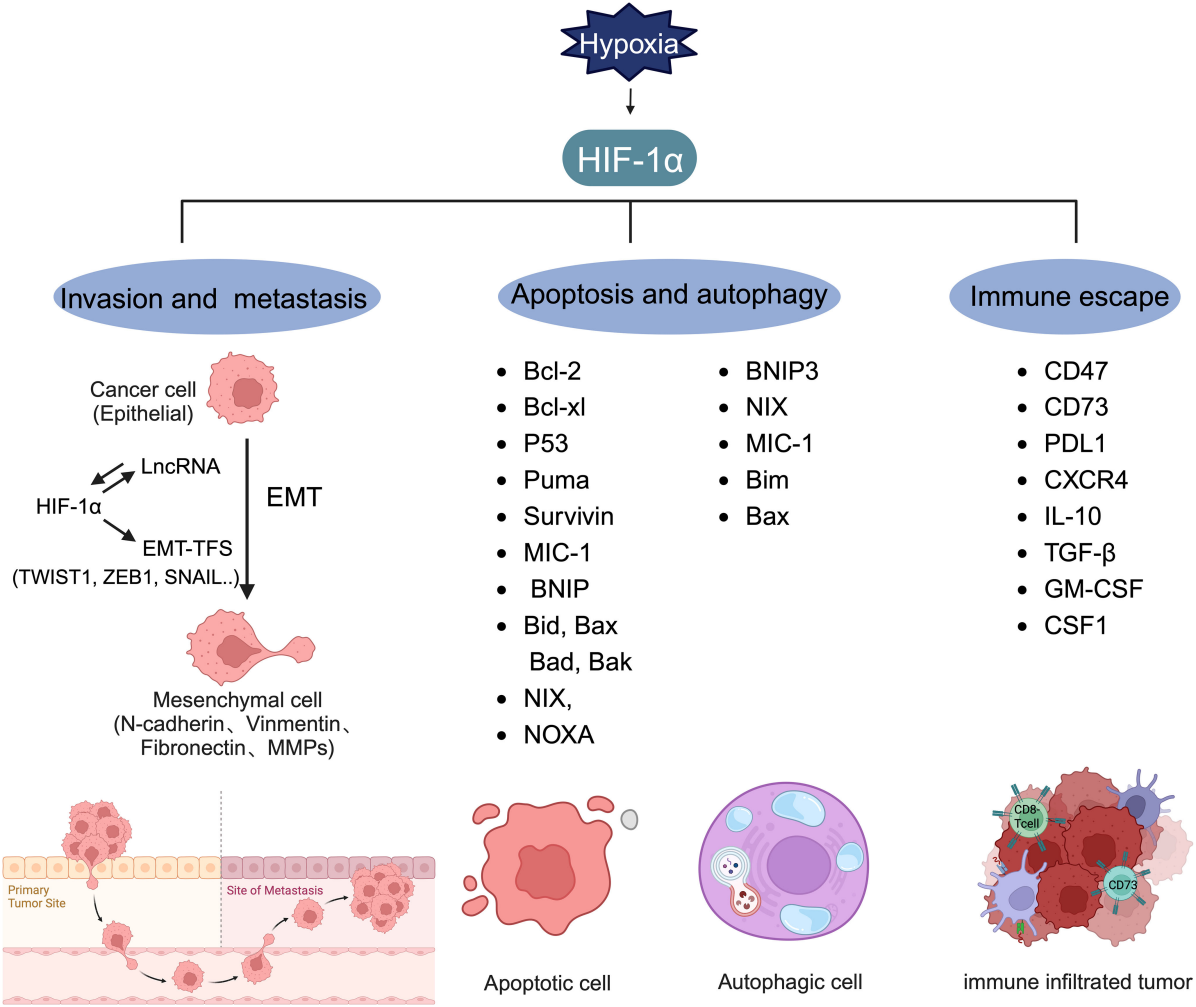
Target genes regulated by HIF-1α. HIF-1α can regulate the expression level of lncRNA and form a mutual activation pathway with lncRNA, thereby promoting the production of EMT-TFs and promoting the process of tumor EMT. HIF-1α plays a key role in inducing the transcription of genes involved in invasion, metastasis, apoptosis, autophagy, and immune escape.

Invasion and metastasis involve the spread of cancer cells from the primary tumor site to distant organs, leading to the formation of secondary tumors (81). Early estimates indicate that nearly two-thirds of cancer-related deaths and three-quarters of BC-related deaths result from metastasis (126). EMT, a process in which epithelial cells convert into mesenchymal cells through specific mechanisms, is a key aspect of tumor metastasis (127, 128). Cancer cells that undergo EMT exhibit enhanced invasive capabilities and resistance to apoptosis. Often, EMT is induced by hypoxia, with HIF-1α overexpression linked to various molecules and pathways (**Figure 5**) (76, 129–132). Research has shown that hypoxia-induced HIF-1α expression leads to the activation of major transcription factors such as TWIST, Snail, Slug, SIP1, STAT3, and ZEB. This activation results in E-cadherin suppression and vimentin induction in BC, with HIF-1α inhibition markedly increasing E-cadherin levels (133). While E-cadherin encourages collective migration of mixed E/M phenotypes by inhibiting TGF-β, TGF-β activation promotes single-cell migration (134). CSF-1, a regulator of EMT, is influenced by hypoxia. HIF-1α induces a mixed E/M phenotype via its target gene CSF-1, facilitating collective migration (135). Additionally, hypoxia has been shown to increase Slug and Snail expression and decrease E-cadherin levels during HIF1-induced EMT through the Notch pathway (136). HIF-1α activates matrix metalloproteinases 1, 2, 9, and 14, aiding in the breakdown of extracellular matrix components and basement membrane degradation, thereby easing cancer cell migration and spread (75, 113, 130, 137, 138). Chemokine receptors 4 (CXCR4) and 3 (CXCR3), associated with invasion, angiogenesis, metastasis, and prognosis, are upregulated by HIF-1-dependent expression, enhancing cell migration and survival during cycling (139, 140). HIF signaling impacts cell extravasation by modulating genes encoding L1 cell adhesion molecules and ANGPTL4, which reduces endothelial cell adhesion (141). He et al. (2020) showed that hypoxia-induced HIF-1α regulates BC cell migration and EMT through the MiR3383p/ZEB2 axis (142). Moon et al. identified MRPL52 as a transcriptional target of HIF-1α, with MRPL52 promoting EMT, migration, and invasion in hypoxic BC cells via the ROS-Notch1-Snail pathway (143).

Recent research indicates that HIF-dependent lncRNAs may contribute to the metastatic phenotype of BC cells. Under hypoxia, lncRNA BCRT1, regulated by HIF-1α transcriptionally, promotes EMT (144). Liu et al. identified HIF-1α as a potential transcription factor for lncRNA HLA complex group 18 (HCG18), with a positive correlation between HCG18 and HIF-1α expression in BC tissue. Knockdown of HIF-1α reduced HCG18 levels in BC cells, and HIF-1α binding to specific HREs in the HCG18 promoter stimulates HCG18 expression. *In vivo* assays showed that decreasing HCG18 expression in MDA-MB-231 cells curbed tumor growth and lung metastasis in xenograft mouse models, highlighting HIF1α’s role as a critical regulator of hypoxia-induced EMT and metastasis (145). Metastasis poses a significant prognostic challenge in BC, and targeting HIF-1α to inhibit BC metastasis presents a viable strategy.

### 3.4 Apoptosis and autophagy

Apoptosis, a genetically controlled form of cell death, is crucial for normal cellular regulation. Cancer cells, however, often evade apoptosis, contributing to chemotherapy resistance or tumor relapse. This evasion involves a complex interplay of proteins and cytokines. Research indicates that HIF-1α exhibits a dual role in apoptosis, capable of both inducing and counteracting it (1). HIF-1α can induce and antagonize apoptosis (**Figure 5**). The proapoptotic effects of HIF-1α involve the regulation of genes such as BNIP, Bid, Bax, Bak, Bad, BNIP3, NIX, and NOXA (146), whereas its antiapoptotic effects are seen in the modulation of Bcl-2, Bcl-xL, and myeloid cell leukemia (Mcl-1) expression (1). In MDA-MB-231 cells treated with paclitaxel, a HIF-1α-dependent alteration in the expression of various pro- and antiapoptotic genes was observed. Under hypoxic conditions, compared to normoxic conditions with paclitaxel, a reduction in proapoptotic gene expression (BAK1, CASP3, CASP8, CASP10, and TNFRSF10A) was noted (147).

HIFs also play a significant role in autophagy, another programmed cell death mechanism (**Figure 5**). In MCF7 cells subjected to radiation, HIF-1α induces autophagy by inhibiting the PI3K/AKT/mTOR/p70 pathway and increases the expression of Mcl-1 and BNIP-3 (148). Mcl-1 participates in the neutralization of proapoptotic proteins, inhibiting cytochrome c release from mitochondria (149), while BNIP-3, a mitochondrial protein in the Bcl-2 family, triggers selective mitophagy by releasing Beclin-1 to initiate autophagy (150, 151). The interplay between autophagy and apoptosis is crucial, especially for inducing cell death in antiapoptotic BC cell lines (152).

### 3.5 HIF-1α in cancer-associated fibroblasts

Cancer-associated fibroblasts (CAFs) interact with tumor cells to promote tumor cell growth and metastasis. CAFs, mainly normal interstitial fibroblasts (NFs), are the most abundant cell type in the stroma of breast cancer (153, 154). In breast cancer tumors, only a small fraction of fibroblasts are quiescent; these fibroblasts are responsible for the structural integrity of the extracellular matrix (ECM) and its nutrient supply and contribute to wound healing (155). However, most fibroblasts exhibit an activated phenotype characterized by producing various extracellular matrix components and paracrine mediators (156). Among the many mechanisms involved in transforming NFs to CAFs, local hypoxia has been proven to drive the differentiation of NFs into activated myofibroblasts by triggering the formation of reactive oxygen species (ROS) (157, 158). Additionally, CAF activation is reversible: chronic hypoxia inactivates CAFs, leading to the loss of contractility, reduction of surrounding extracellular matrix remodeling, and ultimately damage CAF-mediated cancer cell invasion (159). Studies have shown that hypoxia-dependent deletion of PHD2 suppresses tumor growth and reduces the metastatic activity of CAFs (160, 161). Hypoxia inhibited prolyl hydroxylase domain protein 2 (PHD2), resulting in the stabilization of HIF-1α, reduced expression of αSMA and periostin, and decreased myosin II activity. Treatment with the PHD inhibitor DMOG in an orthotopic breast cancer model significantly reduced spontaneous metastasis to the lungs and liver, which correlated with decreased tumor stiffness and fibroblast activation (159). Another study revealed that the loss of PHD2 is associated with normalization of the vasculature, reduced CAF activation, and decreased intravascular invasion of metastases (161). These findings suggest that blocking PHD2 in CAFs may be a novel strategy for inhibiting prometastatic signals in the breast cancer tumor microenvironment.

In fact, CAFs have been shown to regulate metabolic interdependency between cancer cells and their surrounding microenvironment through the action of HIF (162). HIF-1α is involved in regulating biventricular metabolic symbiosis between synthetic metabolic cancer cells and catabolic stromal fibroblasts (163). The enhanced glycolytic rate shown by CAFs is partially dependent on HIF signaling (164). Furthermore, the high-energy metabolic byproducts produced by catabolic CAFs are taken up by tumor cells to support their high anabolic demands. The increased metabolic flux in cancer cells generates ROS, which then propagate throughout the tumor microenvironment and through CAFs to promote HIF-dependent metabolic reprogramming (165).

### 3.6 Immune escape

Tumor immune escape, where cancer cells avoid detection and destruction by the host immune system (166), is facilitated under hypoxic conditions through HIF-1α overexpression(**Figure 5**) (167). HIFs increase CD47 immunoglobulin expression and hinder T cell proliferation and activation by attracting myeloid-derived suppressor cells (28). Changes in the expression of CD47, CD73, and PDL1 in TNBC cells treated with chemotherapy agents like carboplatin, doxorubicin, gemcitabine, or paclitaxel enhance cancer cells’ ability to evade both innate and adaptive immune responses (168). Increased PDL1 mRNA expression in human TNBC cell lines was linked to elevated HIF-1α expression due to endoplasmic reticulum oxidoreductase 1-α (ERO1-α), with ERO1-α knockdown significantly reducing PDL1-mediated T-cell apoptosis, suggesting avenues for therapeutic intervention in hypoxia-mediated immune resistance (169).

Regulatory T cells (Tregs), which suppress immune responses through cytokines and metabolites, play a role in tumor development (170–173). In TNBC, HIF-1α modulates Tregs’ immunosuppressive functions and aggregation by regulating forkhead box P3 (FoxP3) and C-X-C motif CXCR4. HIF-1α enhances FoxP3 expression by binding to HREs and indirectly increases CXCR4 expression; thus, supporting immunosuppression (174). Moreover, M2 macrophages, known for their immunosuppressive capabilities through IL-10 and TGF-β release, are influenced by HIF-1α (175). This factor drives the polarization of tumor-associated macrophages (TAMs) towards an M2 phenotype, creating an immunosuppressive microenvironment. It has been shown that TAMs in TNBC are more likely to adopt an M2 phenotype compared to other BC subtypes (176, 177). HIF-1α, present in BRCA1-IRIS overexpressing TNBC cells, secretes high levels of granulocyte-macrophage colony-stimulating factor (GM-CSF), recruiting and polarizing macrophages towards an M2 phenotype; thus, facilitating immune escape (178, 179). HIF-1α’s ability to polarize TAM to M2 by regulating GM-CSF and macrophage CSF-1 in TNBC underscores its role in promoting immune evasion. HIF can also lead to extracellular acidification by regulating the expression of MCT4, which not only diminishes immune response efficiency but also impacts the efficacy of anticancer drugs (180).

### 3.7 Noncoding RNA regulates HIF-1α

**Figure 6:**
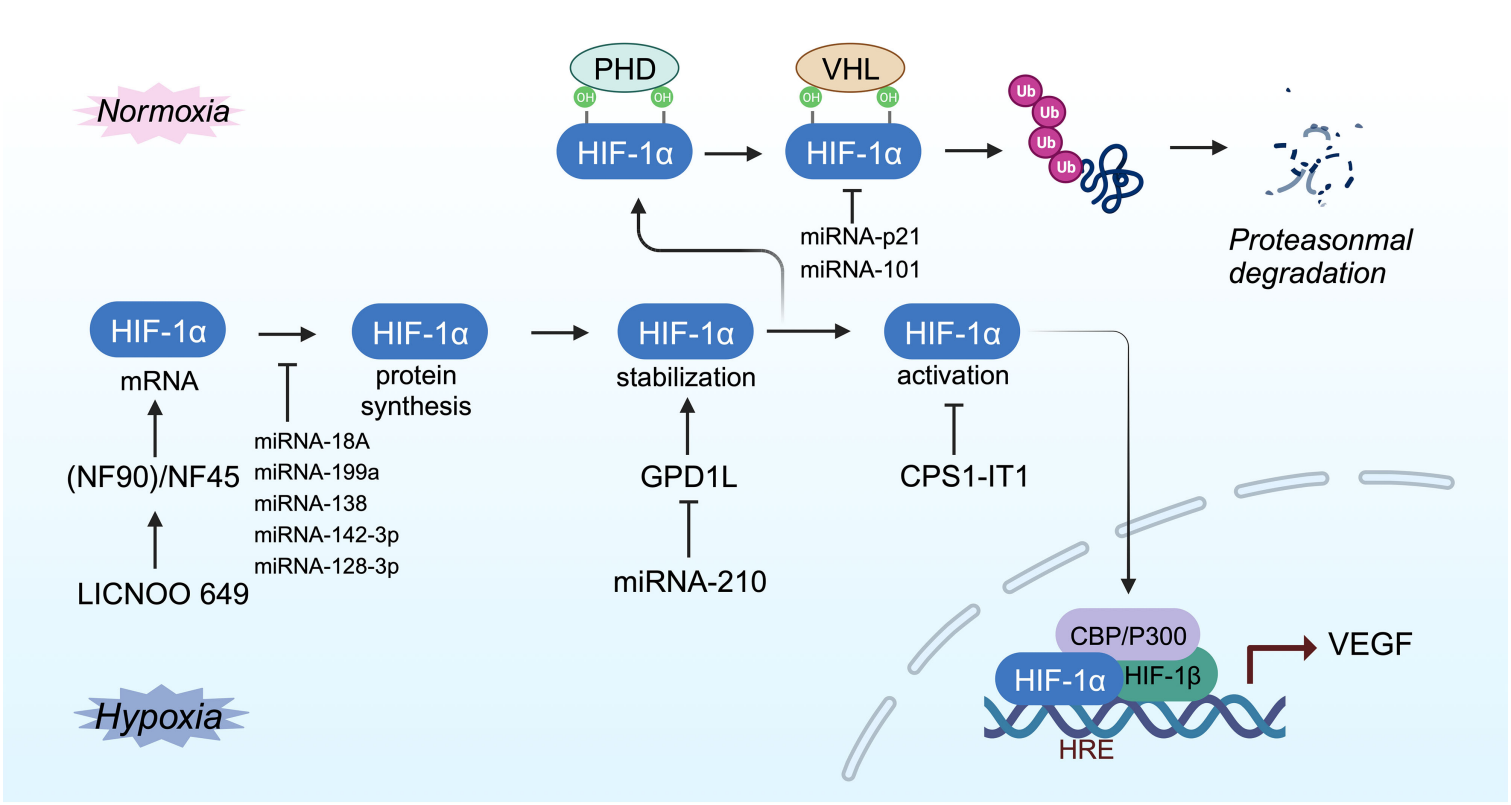
Mechanisms of hypoxia-responsive ncRNA-mediated modulation of HIF-1α activity.

Based on their genomic locations, long noncoding RNAs (lncRNAs) are categorized into five types: antisense, sense, intergenic, intronic, or bidirectional. The location of lncRNAs can be specific to the nucleus, the cytoplasm, or both (25, 181). The expression of lncRNAs is regulated similarly to that of protein-coding RNAs, through mechanisms such as epigenetic modification, gene transcription, and post-transcriptional regulation (182). In tumors, lncRNAs are often abnormally expressed and play roles in the regulation of tumor proliferation, invasion, metastasis, metabolism, angiogenesis, and survival (183, 184). Certain noncoding RNAs can influence tumor-related biological processes by regulating HIF-1α (**Figure 6**) (25, 185, 186). For instance, LINC00649 enhances the stability of HIF-1α mRNA and protein expression by interacting with the nuclear factor 90 (NF90)/NF45 complex (187). MIR210HG specifically impacts triple-negative breast cancer (TNBC) by regulating HIF-1α at the translation level, thereby increasing HIF-1α protein expression and influencing the expression of glycolysis genes (114). MicroRNAs (miRNAs) exert a dual regulatory effect on HIF-1α, both promoting and inhibiting its expression. Saatci et al. identified eight common miRNAs that target HIF-1α, lysyl oxidase (LOX), and ITGA5, with miR-142–3p and miR-128–3p showing negative correlation with the expression of HIF-1α, LOX, and ITGA5. Hypoxia can inhibit miR-142–3p, leading to increased HIF-1α mRNA and protein expression, activating the FAK/Src signaling pathway, and triggering chemotherapy resistance in TNBC (188). However, the regulatory mechanism of miR-142–3p on HIF-1α remains unclear. Overexpression of miR-101 decreases VHL levels, stabilizing HIF-1α and inducing VEGFA expression, ultimately enhancing TNBC invasiveness (189). Thus, noncoding RNA regulates HIF-1α expression in TNBC by maintaining the stability of the HIF-1α protein and regulating the stability and translation level of HIF-1α mRNA.

### 3.8 HIF-1α and drug resistance of breast cancer

**Figure 7:**
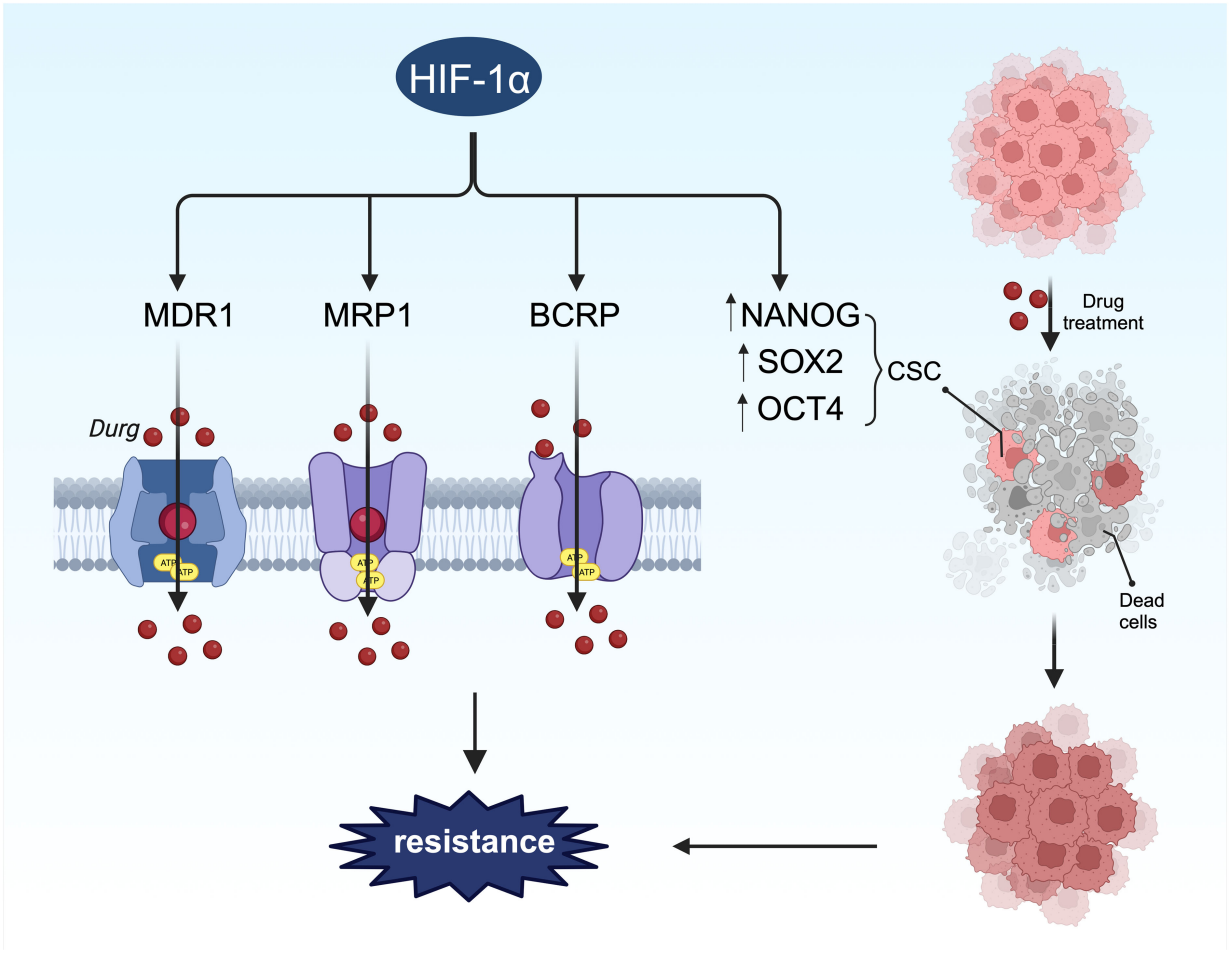
HIF-1α-mediated stemness and drug resistance. On the one hand, HIF-1α can induce drug resistance by regulating stem cell surface markers. On the other hand, HIF-1α promotes chemotherapy resistance through drug resistance-related proteins.

In BC treatment options vary by subtype and include surgery, endocrine therapy, chemotherapy, radiotherapy, and targeted therapy, with drug therapy playing a crucial role depending on the tumor classification. However, drug resistance presents a significant challenge in BC treatment (190–192), often due to inherent or acquired resistance over time (193). Hypoxia is a common characteristic of both primary and metastatic BC (194), with HIF-1α expression in tumor tissues associated with poor prognosis and drug resistance (**Figure 7**) (132). Research indicates that HIF-1α may contribute to resistance against conventional therapies via various signaling pathways, including drug efflux, tumor stem cell enrichment, autophagy, and apoptosis (192, 195), necessitating further investigation into HIF-1α-induced drug resistance mechanisms in BC.

### 3.9 Increased expression of drug outflow pump

Drug efflux transporters such as MDR1, multidrug resistance-associated protein 1 (MRP1), and breast cancer resistance protein (BCRP) are directly regulated by HIF-1α (**Figure 7**), encoded by the ATP-binding cassette (ABC) transporters ABCB1, ABCC1, and ABCG2, respectively. Their promoters contain HREs sensitive to HIF-1α transcriptional regulation (196). The link between these transporters and drug resistance in BC has been extensively studied (197–199), with all three proteins inducing drug resistance by facilitating drug efflux from tumor cells. Studies have shown that hypoxia-induced MDR1 expression can be significantly reduced by inhibiting HIF-1 expression with antisense oligonucleotides (200).In a separate investigation, 41% of BC tumors exhibited increased levels of MDR1, leading to a threefold higher likelihood of chemotherapy failure (201). Taxanes and anthracyclines represent the primary chemotherapy agents for BC treatment. The upregulation of MDR1, also referred to as P-glycoprotein or Pgp, contributes to resistance against taxanes and anthracyclines (202). The activation of HIF-1α enhances the resistance of BC cells to these drugs (147, 203, 204). The genes ABCC1 and ABCG2, which encode MRP1 and BCRP, respectively, possess hypoxia response elements (HREs) upstream of their coding sequences. The deletion of these elements can prevent hypoxia-induced activation (196, 205). MRP1 is elevated in cells with activated HIF-1α, and this effect can be reversed by HIF-1α siRNA, indicating that ABCC1 is a direct target of HIF-1α. Another investigation discovered that HIF-1α binds to the HRE region of the BCRP promoter in LTLTCa cells, with significantly increased binding observed in the presence of CoCl_2_ (206). The expression of BCRP correlated with the degree of drug resistance to irinotecan and topotecan (207). Hence, it is proposed that the expression and stability of HIF-1α can enhance the mRNA and protein levels of MDR1, MRP1, and BRCP, thereby contributing to HIF-1α-mediated drug resistance.

### 3.10 Upregulation of autophagy

Autophagy has been shown to increase resistance to endocrine and cytotoxic drugs in BC (208–210). HIF-1α can activate various molecular mechanisms, such as inducing PTP-PEST expression and activating AMPK, which in turn increases BNIP3 expression. This disrupts the interaction between Beclin-1 and Bcl-2 and induces autophagy, thereby increasing BC cells’ resistance to drugs like adriamycin (211, 212). Additionally, endocrine therapies, including tamoxifen and flurvist, have been found to increase autophagy markers in drug-resistant BC cells (213, 214). Thus, HIF-1α plays a role in inducing BC resistance to both endocrine and cytotoxic drugs by upregulating autophagy.

### 3.11 Inhibition of apoptosis

Apoptosis, leading to cell death, is a crucial outcome of most cancer treatments. Cancer cells, however, have developed mechanisms to evade apoptosis, contributing to resistance to chemotherapy or recurrence of tumors (215). This evasion involves various proteins and cytokines, including the Bcl-2 family, apoptosis inhibitor proteins, and the caspase family, along with cytochrome c and proteases (146). HIF-1α plays a direct role in the regulation of apoptosis, exhibiting both proapoptotic and antiapoptotic effects (146). The proapoptotic actions of HIF-1α include the downregulation of proapoptotic members of the Bcl-2 family, such as BNIP3, NIX, and NOXA, while its antiapoptotic effects involve increasing the levels of antiapoptotic proteins like Bcl-2, Bcl-xL, and Mcl-1, and decreasing the levels of proapoptotic proteins such as Bid, Bax, and Bak (216–218). In breast cancer (BC) cells, alterations in apoptotic activity following HIF-1α activation are associated with increased drug resistance (147, 219, 220), though the precise mechanisms warrant further investigation.

### 3.12 Maintenance of the dryness of cancer cells

Stem cells play a significant role in BC, driving tumor progression and metastasis, and displaying inherent resistance to chemotherapy and radiotherapy (221, 222). These cells can enhance drug efflux, increase drug metabolism in the tumor, activate survival pathways like Notch and Hedgehog, and dampen apoptosis signaling, all contributing to chemoresistance (223). HIF-1α directly upregulates genes such as NANOG, SOX2, KLF4, and OCT4, which inhibit differentiation and maintain a stem cell-like phenotype (203, 224). Furthermore, HIF-1α supports the survival of breast cancer stem cells (BCSC) by inducing ROS-dependent expression of HIF-1α and HIF-2α, leading to HIF-mediated expression of IL-6, IL-8, and MDR1. Exposure of MDA-MB-231, SUM-149, and SUM-159 cells to paclitaxel increased the percentage of ALDH+ cells, indicative of stem cell characteristics, by twelvefold *in vitro* and *in vivo* (203). Therefore, HIF-1α activation promotes the proliferation and enrichment of tumor stem cells, contributing to drug resistance.

### 3.13 Strategies to overcome HIF-1α-dependent drug resistance

To overcome HIF-1α-dependent drug resistance, strategies involve directly or indirectly targeting HIF-1α with inhibitors (225). Direct inhibitors of HIF-1α target protein-protein/DNA interactions, impacting DNA binding, transcriptional activity of HIF-1α, heterodimerization with HIF-1β, and interactions with transcriptional coactivators (226–230). Indirect inhibitors aim to downregulate HIF-1α transcription or translation, reduce HIF-1α stability, or prevent its degradation, offering potential pathways to mitigate drug resistance in cancer treatment.

### 3.14 Breast cancer therapy targeting HIF-1α

Numerous studies have highlighted the potential of HIF-1α inhibitors and compounds targeting the HIF-1α pathway, demonstrating their effectiveness *in vitro* (62, 81)(**Table 2**). For instance, treatment of MDA-MB-231 cells with the PHD inhibitor molidustat, which stabilizes HIF, resulted in reduced viability, growth, clone formation, cell cycle arrest, and increased chemosensitivity, indicating potential anticancer activity of HIF (249) Among 68 newly synthesized arylformamide compounds, compound 30 m showed the most potent inhibitory activity against HIF-1α with minimal cytotoxicity, effectively reducing hypoxia-induced HIF-1α protein accumulation (81, 250). However, clinical trials have failed to show the effect of HIF-1α inhibitors in BC treatment (81, 251)(**Table 3**). Preliminary data published in 2013 showed some clinical efficacy of digoxin in patients with stages I–III BC. However, the treatment window of the drug is narrow; serum levels exceeding 1–2 nM produce significant side effects. So far, no follow-up data are available (192, 252). The differential expression of HIF-1α and HIF-1α inhibitor monotherapy may be the factors limiting the efficacy of anti-HIF-1α therapy (1). Yu et al. discussed the lack of clinical effectiveness of HIF-1α inhibitors (230). First, although both HIF-1α and HIF-2α are involved in cancer progression, most inhibitors have targeted only HIF-1α. Therefore, the role of HIF-2α in drug resistance needs to be understood. Second, patient selection contributes to the success of clinical trials. Accuracy in the measurement of tumor HIF-1α may help to better distinguish responders from non-responders. Finally, although hypoxia varies between and within different BC subtypes, evidence suggests that the hypoxia of the patient’s tumor itself is different (253). This heterogeneity may be the main determinant of the overall drug response. Considering the limitations of HIF-1α inhibitors, a combination of HIF-1α inhibitors with chemotherapeutic drugs or other agents may improve outcomes. A preclinical study indicated that digoxin enhances the sensitivity of triple-negative breast cancer cell lines to paclitaxel and gemcitabine *in vivo* (203). Interestingly, molidustat has been found to enhance the cytotoxicity of gemcitabine in MDA-MB-231 cells (249). However, currently, the related combination therapy of BC is in the preclinical stage, and thus, a clear conclusion cannot be drawn. The latest HIF-1α drug delivery system is based on nanocarriers that can improve targeting specificity, overcome solubility problems, reduce drug toxicity, and maintain safe drug concentrations. Furthermore, the mode of drug administration also affects the efficacy of HIF-1α-related drugs and should be investigated in future research (81). In conclusion, the results on BC cell lines show that aspects, such as comparison of HIF-1α and HIF-2α inhibition, double vs. single isomer inhibition, different statuses of hormone receptors, metastasis, and other unexplored issues, should be considered. Thus, we need to understand the role of HIFs in BC before targeting them for clinical application (55).

**Table 2:** HIF-1α inhibitors under investigation in BC.

Compound	Mechanism	Type of study	Model	References
KC7F2	Decrease HIF-1a protein accumulation	*In vitro*	MCF-7	(231)
Quercetin	Inhibiting HIF-1a protein accumulation	*In vitro*	SkBr3	(232)
LXY6006	Inhibit HIF-1a nuclear accumulation	*In vitro*	T47D, MDMBA-231, MX-1	(233)
Aminoflavone	AF inhibits HIF-1α expression	*In vitro*	Mouse model (MCF-7)	(234)
7-Hydroxyneolamellarin A	Inhibit HIF-1a protein accumulation	*In vivo*	Mouse model (4T1)	(235)
Methylalpinumisoflavone	Inhibits HIF-1 activation by blocking the induction of nuclear HIF-1α protein	*In vitro*	T47D, MDAMB-231	(236)
Cardenolides	Inhibited HIF-1 transcriptional activity dose-dependentl	*In vivo*	MCF-7	(237)
PX-478	Suppresses HIF-1a levels	*In vitro*	Mouse model (MCF-7)	(238)
DJ12	Decrease HIF-1a transactivation and DNA binding	*In vitro*	MDA-468, ZR-75, MD435	(239)
Isoliquiritigenin	Isoliquiritigenin inhibits the expression of HIF-1α by inhibiting the PI3K/Akt and NF- κB signaling pathways, thereby inhibiting the expression of VEGF and the metastasis of TNBC	*In vitro*	MDA-MB-231 cells	(240)
Cardamonin	Cardamonin inhibits the transcriptional level of HIF-1α by inhibiting the mTOR/p70S6K pathway, reducing the level of HIF-1α protein, thereby enhancing mitochondrial oxidative phosphorylation and reducing glucose uptake and lactate production.	*In vitro*	MDA-MB-231 cells	(241)
Nanoliposomalechinomycin	The activity of HIF-1 can be inhibited by directly inhibiting the transcriptional activity of HIF-1α and effectively blocking the binding between HIF-1 and HRE.	*In vivo*	MDA-MB-231 breast cancer mice and SUM-159 breast cancer mice	(242)
Melittin	Melitin inhibits HIF-1α expression at the transcriptional level mainly by inhibiting NF-kB expression	*In vitro*	MDA-MB-231 cells	(243)
As4S4 nanoparticles	As4S4 nanoparticles reduce the transcription level of HIF-1α by scavenging ROS and inhibit the metastasis of TNBC.	*In vivo*	4T1 breast cancer mice	(244)
Sanguinarine	Sanguinarine promotes proteasomal degradation of HIF-1α by inactivating STAT3 under hypoxia and hinders breast cancer growth *in vivo*	*In vitro*	MDA-MB-231 cells	(245)
Ganetespib	Ganetespib promotes the degradation of HIF-1α protein, reduces the levels of HIF- 1α protein and target gene proteins and controls angiogenesis, metabolism, invasion and metastasis in TNBC mice.	*In vitro*	MDA-MB-231 cells	(246)
Acriflavine	Inhibition of TNBC premetastatic niche formation by targeting HIF-1α	*In vitro*	MDA-MB-232 cells	(247)
Diallyl Trisulfides	DATS inhibits the synthesis of HIF-1α protein by inhibiting the translation level of HIF-1α, thereby reducing the transcriptional activation of downstream target genes L1CAM, snail, slug, VEGF and MMP-2, thereby inhibiting the metastasis of TNBC.	*In vitro*	MDA-MB-233 cells	(248)

**Table 3:** HIF-1α related clinical studies in BC.

Drug	Main Outcomes	Conditions	Clinical Phase	Study Type	Status	NCT Number
Digoxin	There was not enough data to analyze HIF-1alpha expression because of the limited tumor samples	Breast Cancer	II	Interventional	Completed	NCT01763931
Bevacizumab, docetaxel	The rate of serious adverse events is about 18.06% and the rate of other adverse events is 98.61% in total	Breast Cancer	II	Interventional	Completed	NCT00559754
Paclitaxel plus bevacizumab	There was no significant difference between HIF-1alpha polymorphism and longer PFS in patients treated with paclitaxel and bevacizumab	Metastatic Breast Cancer	I	Observational	Completed	NCT01935102
Vinorelbine	Metronomic dosing of oral vinorelbine in HR+/HER2- MBC as first-line CT after failure of endocrine therapies showed only limited benefit in patients	Metastatic Breast Cancer	II	Interventional	Completed	NCT03007992

## 4 Prospect

HIF-1α, an important transcription factor under hypoxic conditions, plays a crucial role in the growth and development of the body as well as various physiological and pathological processes. HIF-1α induces the expression of numerous genes related to angiogenesis, growth and survival, invasion and metastasis, glucose metabolism, epithelial–mesenchymal transition (EMT), immune evasion, and resistance to various cancer treatments. The overexpression of hypoxia-inducible factor-1α (HIF-1α) is also closely related to drug resistance and prognosis in breast cancer patients. Therefore, regulating the activity of HIF-1α may be a breakthrough in treating many diseases. Upregulating the activity of HIF-1α can increase cell survival under hypoxic conditions and enhance angiogenesis in hypoxic tissues. Conversely, HIF-1α inhibitors can block angiogenesis and reduce the survival rate of hypoxic or inflammatory tissues. This article elaborates on the structure and function of HIF-1α, its mechanism of action in developing breast cancer, and drug resistance mechanisms. This review also summarizes strategies to overcome HIF-1α-dependent drug resistance and the current status of targeted HIF-1α therapy for breast cancer.

However, the complex interactions among multiple pathways involving HIF-1α pose greater challenges for its clinical application as an inhibitor. A deeper understanding of the intricate interactions between oxygen tension, the microenvironment, receptor expression, and HIF-1α expression is needed. This not only facilitates the development of new drug combinations but also aids in the discovery of novel drug targets for breast cancer treatment. Currently, drugs targeting HIF-1α are mainly focused on preclinical research, and their actual clinical effects, patient tolerance, dosing regimens, and other factors require further evaluation and validation. Additionally, drug delivery and efficacy are limited by factors such as tumor acidosis, hypoxic microenvironments, and elevated interstitial fluid pressure within tumors. Therefore, there is a need to develop more suitable drug carriers and delivery systems to enhance therapeutic outcomes. In conclusion, more comprehensive and in-depth research is required on HIF-1α and the pathways it mediates. Although current clinical trials have not yet demonstrated satisfactory results for HIF-1α inhibitors as monotherapy in breast cancer treatment, with continuous and in-depth research on the role of HIF-1α in cancer development, it is believed that targeted HIF-1α therapeutics will bring new hope to breast cancer patients in the near future.

## Author contributions

ShiZ: Validation, Writing – original draft. CC: Validation, Writing – review & editing. HH: Visualization, Writing – review & editing. ZZ: Supervision, Writing – review & editing. FZ: Conceptualization, Funding acquisition, Writing – review & editing. ShuZ: Conceptualization, Funding acquisition, Writing – review & editing.

## Glossary

**Table:**

BC	Breast cancer
EMT	Epithelial-mesenchymal transition
HIF	Hypoxia-inducible factor
HIF-1α	Hypoxia-inducible factor-1α
HIF-2α	Hypoxia-inducible factor-2α
HIF-3α	Hypoxia-inducible factor-3α
HIF-1β	Hypoxia-inducible factor-1β
bHLH- PAS	Basic helix-loop-helix/Per-ARNT-Sim
TADs	Transactivation domains
C-TAD	C-terminal transactivation domain
N-TAD	N-terminal transactivation domain
CBP/p300	cAMP response element-binding protein and p300 protein
ODDD	Oxygen-dependent degradation domain
NLS	Nuclear localization signals
PHDs	Prolyl hydroxylase domain-containing proteins
FIH	Factor inhibiting HIF-1α
VHL	Von Hippel–Lindau protein
Ub	Ubiquitin
HRE	Hypoxia response element
TNBC	Triple negative breast cancer
NF-κB	Nuclear factor-κB
IKK	Inhibitor of NF-κB kinase
TNF	Tumor necrosis factor
IL1	Interleukin 1
VEGF	Vascular endothelial growth factor
VEGFR	Vascular endothelial growth factor receptor
HGF	Hepatocyte growth factor
VCAM1	Vascular cell adhesion molecule 1
BRK	Breast tumor kinase
C1QBP	Complement C1q binding protein
BDNF	Brain-derived neurotrophic factor
ROS	Reactive oxygen species
ANGPTL4	Angiopoetin-like 4
OXPHOS	Oxidative phosphorylation
GLUT	Glucose transporter
HK2	Hexokinase 2
PFK1	6-phosphofructokinase1
ALD	Aldolase
ENO	Enolase
GAPD	Glyceraldehyde-3-phosphate dehydrogenase
PGK	Phosphoglycerate kinase
PK	Pyruvate kinase
LDHA	Lactate Dehydrogenase A
PKM2	Pyruvate kinase isozymes M2
PDK1	Pyruvate dehydrogenase kinase 1
PDH	Pyruvate dehydrogenase kinase
MCT	Monocarboxy latetransporter
TCA	Tricar boxylic acid
Twist1	Twist-related protein 1
STAT3	Signal transducer and activator of transcription 3
ZEB	Zinc finger E-box binding homeobox
TGF-β	Transforming growth factor-β
CSF-1	Colony-stimulating factor 1
MMP	Matrix metalloproteinase
CXCR4	C-X-C chemokine receptor type4
CXCR3	C-X-C chemokine receptor type3
BNIP	BCL2/adenovirus E1B 19 kDa interacting protein
Bid	BH3 interacting domain death agonist
BAX	Bcl-2-associated X protein
BAK	BCL2 antagonist/killer
Bad	BCL2 associated agonist of cell death
BNIP3	B cell lymphoma 2 interacting protein 3
NIX	NIP3-like protein X
BCL2	B-cell lymphoma 2
Bcl-XL	B-cell lymphoma 2-extra-large protein
BNIP3L	BNIP3-like protein
PI3K	Phosphoinositide 3-kinase
AKT	Protein kinase B
mTOR	Mechanistic target of rapamycin
BIM	BCL2 like 11
Tregs	Regulatory T cells
PDL1	Programmed cell death ligand 1
ERO1	Endoplasmic reticulum oxidoreductin 1
FOXP3	Forkhead box protein 3
IL10	Interleukin 10
GM-CSF	Granulocyte-macrophage colony-stimulating factor
NF45	Nuclear factor 45
NF90	Nuclear factor 90
LOX	Lysyl oxidase
ITGA5	Integrin alpha 5
MDR1	Multidrug resistance gene 1
MRP1	Recombinant multidrug resistance associated protein 1
BCRP	Breast cancer resistance protein
ELP3	Elongator complex protein 3
PAK1	P21-activated protein kinase
AMPK	AMP-activated protein kinase
ATG5	Autophagy-related gene 5
CSC	Cancer stem cell
Oct4	Octamer transcription factor 4
SOX2	SRY-box transcription factor 2
NANOG	Nanog homeobox
KLF4	Kruppel-like transcription factor 4.
